# Associations between dialysis modality and adherence to immunosuppression after kidney transplantation—A single-center study

**DOI:** 10.1371/journal.pone.0317435

**Published:** 2025-01-24

**Authors:** Fernanda Ortiz, Aino Salonsalmi, Ilkka Helanterä

**Affiliations:** 1 Helsinki University Hospital, Abdominal Centre, Nephrology and University of Helsinki, Helsinki, Finland; 2 Helsinki University Hospital, Abdominal Centre, Transplantation and Liver Surgery, and University of Helsinki, Helsinki, Finland; Warren Alpert Medical School of Brown University: Brown University Warren Alpert Medical School, UNITED STATES OF AMERICA

## Abstract

**Background:**

Patients with end-stage kidney disease often prefer home-based dialysis due to higher self-efficacy, which relates to improved medical treatment adherence. Kidney transplantation (KT) success depends on adhering to immunosuppressive medication post-transplant.

**Objectives:**

To investigate whether adherence post-kidney transplantation (KT) and patients’ attitudes toward immunosuppression were influenced by their prior dialysis type modality. Additionally, the study examined if adherence and patient’s attitudes towards immunosuppression are associated with kidney graft survival.

**Methods:**

This cross-sectional single-center study included 201 KT patients. Adherence was assessed using BAASIS and the coefficient of variation of calcineurin inhibitors (COV-CNI). Patient attitudes towards medication were evaluated using the Q-method. Pill burden, comorbidity score and HRQoL and medication complexity, were scored. Cox regression was applied to determine KT survival outcomes over a 14-year follow-up period (until Dec 2021).

**Results:**

Prior dialysis modality was not associated with adherence to immunosuppression post-KT evaluated by BAASIS on average 4.7 years post-KT. Previous in-center hemodialysis patients had a higher CNI-COV (p = 0.011). The Q-sort analysis identified fully adherent patients linked to profile 1 (organized, resilient) whereas profile 2 patients were more careless. Patients linked to profile 3 (challenging, nervous) had higher education, a higher pill burden, and experienced more immunosuppression side effects. Death-censored graft loss increased by 7.6% with each additional pill, quadrupled if one dose of immunosuppression was missed, and increased by 2.9% for each point of COV-CNI rise.

**Conclusions:**

Adherence to immunosuppression post-KT using BAASIS was not associated with prior dialysis type, despite in-center hemodialysis patients showing the highest COV-CNI. Taking COV-CNI into account, managing missed doses of immunosuppressants, and exploring patient attitudes could potentially enhance adherence and consequently improve KT survival.

## Introduction

Patients undergoing home dialysis generally demonstrate better adherence to medical treatment compared to those receiving in-center dialysis. This is often credited to improved social functioning and adherence to dialysis procedures, medication intake, fluid management, and lifestyle adjustments, all of which are associated with mortality risks [1]. Successful home-based dialysis heavily relies on patient self-management, encompassing five critical aspects: communication, involvement in decision-making, self-care tasks, integration of self-management practices, and adherence to the prescribed treatment regimen [2]. Furthermore, health literacy among home dialysis patients seems higher compared to individuals undergoing in-center hemodialysis or those with non-dialysis-dependent CKD [3]. In addition, patients undergoing home dialysis are in general higher educated [4] and higher education has been associated with better adherence [5].

Despite adequate dialysis, studies suggest that kidney transplant recipients experience a better health-related quality of life (HRQoL) than dialysis patients [6, 7]. Graft loss has been associated with lower HRQoL [8]. Effective administration of immunosuppressive treatment is crucial for maintaining transplanted kidney function; however, nearly one-third of recipients exhibit non-adherence, significantly contributing to both survival and HRQoL [9]. Consistent adherence to lifelong immunosuppressive therapy is pivotal for transplant survival success [6, 7] Patient personality has been proposed to influence adherence behavior in chronic diseases [10] and transplantation settings [11]. Therefore, a comprehensive approach is essential to understand the multifaceted dimensions of medication adherence that influence patient behavior.

Various methods are utilized to assess non-adherence, such as patient surveys, electronic pillboxes, pharmacy data, and drug concentration measurements in the blood [12]. Nevertheless, a standardized method to evaluate non-adherence is lacking, posing challenges in correlating, and comparing these approaches [2]. Combining these tools could potentially enhance the reliability and validity of collected data [13].

Although high self-efficacy has been related to better adherence, this has not yet been studied if it applies to transplant patients previously treated with home dialysis, including home hemodialysis and peritoneal dialysis. Therefore, we hypothesize that previous home dialysis patients have high adherence to immunosuppression after transplantation. The main aim of this study was to examine if dialysis modality is associated with post-kidney transplantation immunosuppression adherence utilizing multiple methodologies: the BAASIS questionnaire for adherence and calcineurin exposure. A further aim was to examine if dialysis modality is associated with personality type assessed by Q-sort analysis and if the personality type is associated with medication adherence. We also explored their correlation with patients’ medication attitudes, HRQoL, and medication treatment complexity. Lastly, we examined the associations of non-adherence t with graft survival.

## Methods

### Design, study setting, and participants

**Fig 1:**
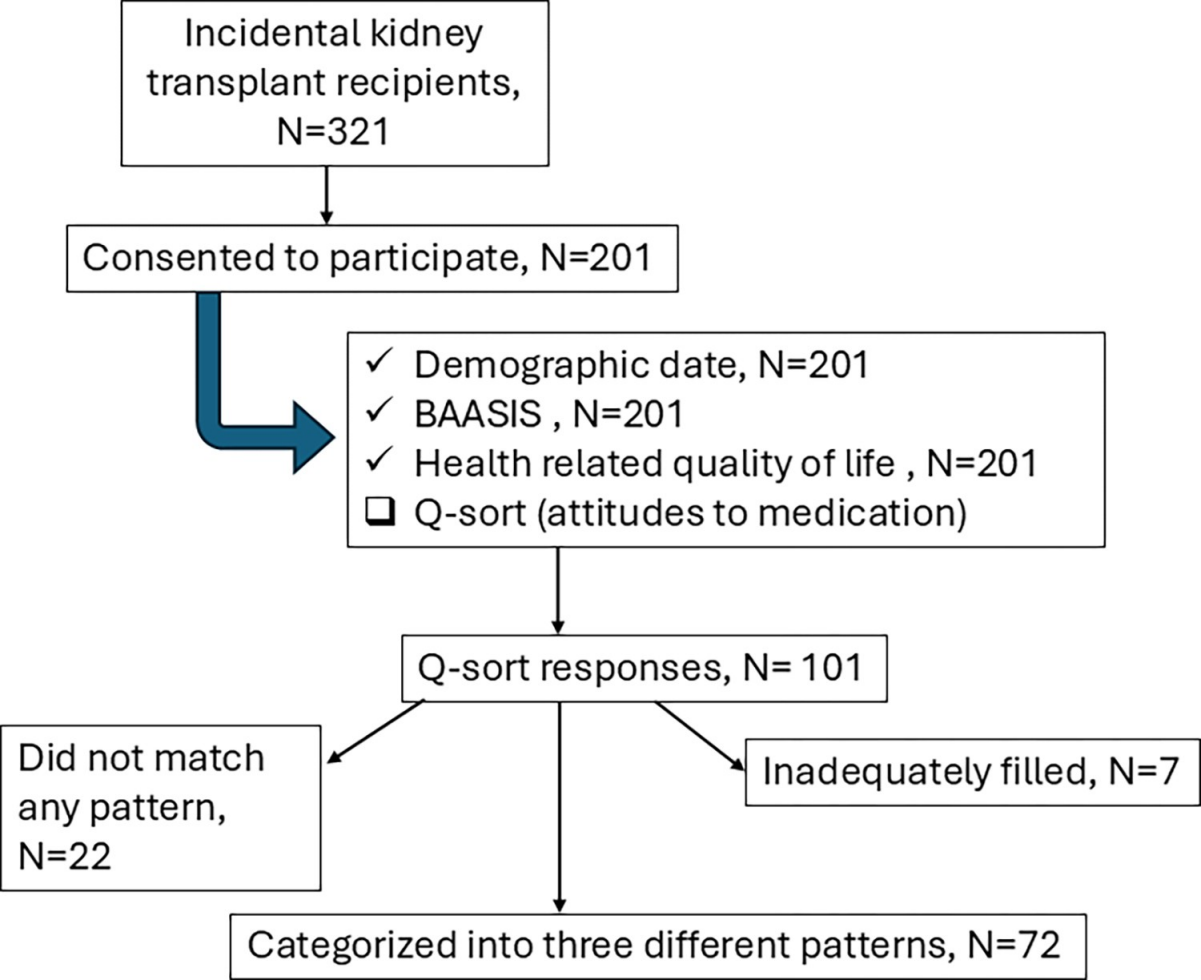
Participants flow chart.

Patients who underwent kidney transplantation (KT) at Helsinki University Hospital after January 1, 2004, and were under follow-up care were included in this single-center cohort cross-sectional survey study. Between October 1^st^, 2012, and March 31^st,^ 2013, adult patients with a functioning graft attending the outpatient clinic received invitations by mail. Among 323 patients, 201 (62%) returned questionnaires with written informed consent, therefore recruited for this investigation. These participants were monitored until graft loss, death, or December 31, 2021. Participants flowchart is displayed in Fig 1.

### Measurements

Data on prescribed medication and additional medical details were gathered from patient charts when the questionnaires were completed. This information encompassed demographics, educational background (basic education, high school, university, other), pre-and post-transplant employment status (working, retired, studying), annual kidney function using a creatinine-based glomerular filtration rate estimation (eGFR) post-transplantation, calcineurin inhibitor concentrations, occurrences of acute rejection, and both graft and patient survival.

### Measurement of adherence, attitudes to immunosuppressive medication, and HRQoL

The BAASIS questionnaire ® [14] is a widely use tool to evaluate adherence to immunosuppression. It consists of questions focused on four dimensions: taking drug holidays, timing of medication intake, regularity of medication intake, and dose reduction. The version applied in this study included the visual analog scale (VAS), where the patient rates his/her medical treatment success performance from 0 to 100. Permission to use the BAASIS questionnaire was obtained from the University of Basel. We considered non-adherence as any deviation in taking, timing, or dosing.

Adherence to immunosuppression was also investigated by calculating the calcineurin inhibitor’s coefficient of variation (COV-CNI). The calculation was based on all trough-level measurements from the first year after transplantation until the end of follow-up. We chose 30% as the cutoff for low and high variability based on previous studies [15]. Of note, immunosuppressive medication is fully reimbursed by the national health insurance in Finland.

The medication complexity was calculated with the medication regimen complexity index (MRCI), a numerical value based on the number of pills, number of medications, frequency, and types of administration daily. This index does not have a maximum number, but the higher the score, the more complex the medical treatment. The original MRCI has been adapted for KT patients [6, 16]. Patients´ comorbidity was evaluated with the recipient risk score (RRS) [17].

Patient attitudes to medication were studied with the Q-methodology [18, 19]. Q-methodology has been associated with medication adherence in transplant patients [20]. The chosen 38 statements are based on previous investigations in kidney transplant patients found in the literature. We asked the participants to rank-order them based on to what extent they agreed or disagreed with each topic. This is referred to as ‘Q sorting.’ The statements are matters of opinion only (not fact), and this is what brings subjectivity into the picture. The participants were asked to explain the reasons why they picked to most agreed and least agreed statements in their own words.

In case the patient found this task difficult, a research nurse assisted them. These individual rankings (or viewpoints) are then subject to factor analysis. The patterns of responses provided by the factor analysis were categorized along with the five-factor model of personality (neuroticism, extraversion, openness to experiences, agreeableness, and conscientiousness), which has been suggested to influence adherence behavior in patients with chronic disease [10] and transplantation [11].

HRQoL was investigated with the 15D instrument, a generic 15-dimensional, standardized, and self-administered measure of HRQoL [21]. This instrument has been used before in KT patients [4]. The questionnaires and MRCI scoring sheets are available in S1 Questionnaires.

**Fig 2:**
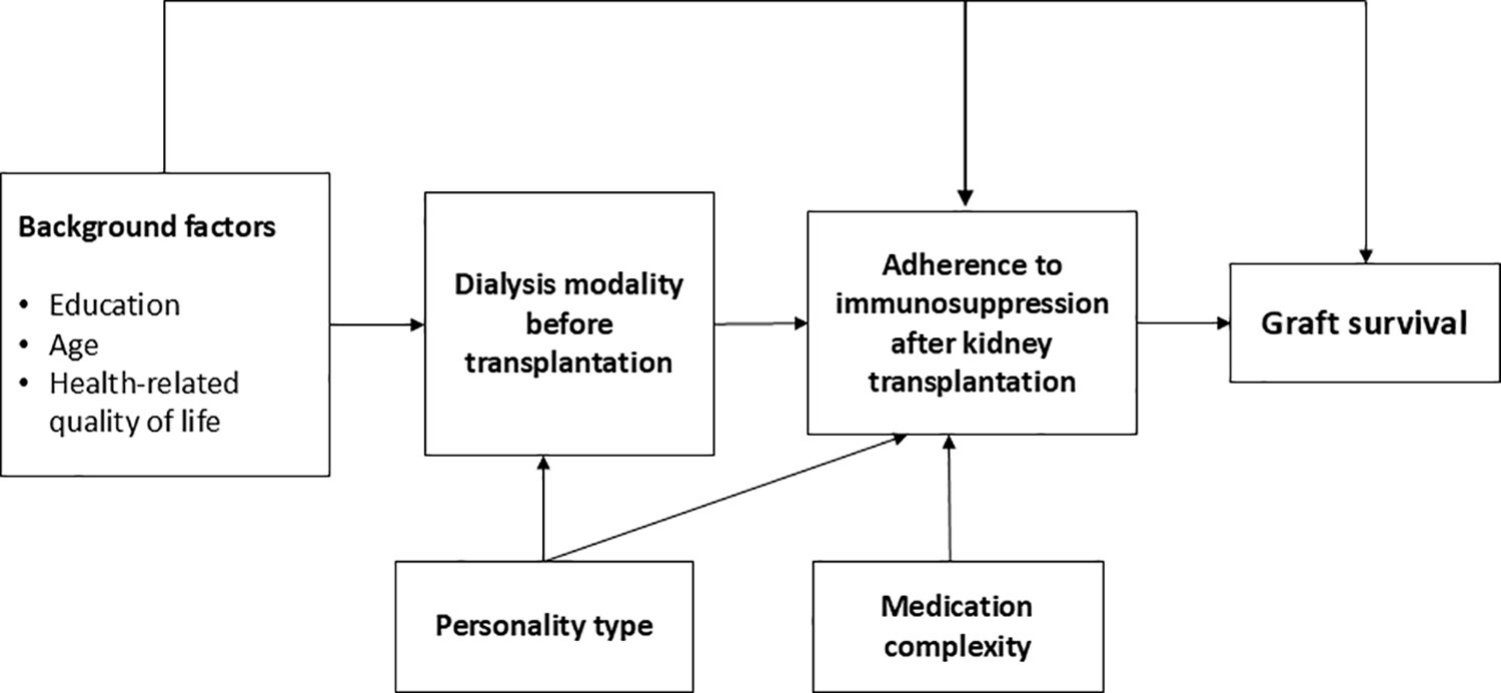
Directed acyclic graph of the assumed associations between dialysis modality before transplantation, adherence to immunosuppression medication after kidney transplantation and background factors.

The assumed associations between dialysis modality before transplantation, adherence to immunosuppression medication after kidney transplantation and background factors are described in the directed acyclic graph (Fig 2).

### Statistical analysis

Descriptive statistics are presented as the mean and 95% CI of the mean. Continuous variables were tested for normality with Shapiro-Wilk, analysis of histograms, and Q-Q plots. Pill number, medicines number, MRCI, time on dialysis, eGFR at follow-up, 15D score, and VAS did not follow a normal distribution and therefore were log10 transformed. In case the variables did not follow a normal distribution after log transformation, a non-parametrical test was applied for group comparison. The χ^2^ test was applied for categorical variables with Bonferroni correction.

Q-methodology was evaluated with factor analysis and varimax rotation of the rankings of statements provided by each participant. By this method, a limited number of corresponding patterns were revealed in the way participants sorted the statements. Correlation between individual rankings of statements indicated similar viewpoints. Three different patterns of attitudes towards immunosuppression were recognized, hereafter called profiles one to three.

Survival analysis was examined with Kaplan-Meier and the Breslow test was used for differences between kidney graft survival and the profiles or BAASIS-results. Cox regression analysis was used to investigate the associations of each item included in the multimethodical evaluation of non-adherence with graft survival and death-censored graft survival. The model was adjusted for pill number, COV-CNI, missing any dose, or failure to take medications on time, Q-sort factors, age, previous acute rejection, and HRQoL. The risk started at the time of transplantation.

Linear regression was used to determine if the 15D scores differed between dialysis modalities after adjustment for gender and age. If there were up to three missing answers, the missing data were imputed by regression models with age, sex, and the responses to the other dimensions as explanatory variables. The use of this methodology has been previously used. [4] A p-value <0.05 was considered statistically significant. Statistical software SPSS 19 and R3.0.1 (IBM, Armonk, NY, USA) was used for the analysis.

This research was approved by the Ethical Committee of the Helsinki University Hospital (HUS/146/13/03/01/12) to ensure that the work is done in accordance with the Declaration of Helsinki and the Declaration of Istanbul.

## Results

Pre-transplantation, 38.8% underwent peritoneal dialysis (PD), and 24.9% received home hemodialysis (HHD) with personalized training from a nephrology nurse, lasting one to six weeks. Contrastingly, patients previously treated in in-center hospital dialysis (ICHD: 35.8%) did not receive personalized self-care dialysis training. Those declining participants had similarities in age, sex, dialysis duration, and dialysis modalities to the study participants.

The interval from KT to the survey averaged 4.7 years, extending up to 14 years post-KT. Of the respondents, 50 individuals died with a functioning graft, and 29 resumed dialysis post-survey completion. At participation, the mean age was 57 years (95% CI 55–58), and the mean eGFR was 57 ml/min (95% CI 54–60). Approximately 64% of patients received pre-transplant home dialysis training: HHD (24.9%), CAPD (13.4%), and APD (25.4%). One patient received a transplant without being previously on dialysis (0.5%), while 35.8% of ICHD patients lacked personalized home dialysis training. Participant demographics are outlined in Table 1.

**Table 1:** Results are expressed as means (95% CI) unless otherwise indicated. Statistically significant differences are shown in bold. Pre-emptive Tx is omitted in this table (N = 1). HRQoL was measured with a 15D instrument. DM: diabetes mellitus. CGN: chronic glomerulonephritis. ADPKD: autosomal dominant polycystic kidney disease. CIN: chronic interstitial nephritis. CKD: chronic kidney disease. RRS: recipient risk score for comorbidities.

	*CAPD* *(N = 27)*	*APD* *(N = 51)*	*HHD* *(N = 50)*	*ICHD* *(N = 72)*
*Age at response*	57 (51–62)	55 (52–58)	58 (55–60)	58 (55–61)
*Sex, M/F (N and %)*	16/11 (59/41)	30/21 (59/41)	40/10 (80/20)	38/34 (53/47)
*Time on dialysis, months*	26 (14–38)	22 (18–26)	24 (17–31)	31 (26–36)
*RRS*	4.3 (3.8–4.7)	4.1 (3.8–4.4)	4.1 (3.8–4.3)	4.3 (4.0–4.5)
*eGFR at response date*	57 (49–56)	59 (52–65)	52 (46–59)	59 (53–65)
*eGFR at end of FU*	57 (48–66)	59 (52–65)	52 (46–87)	59 (53–65)
*Acute rejection, in %*	15	20	14	11
*HRQoL*	0.87 (0.81–0.91)	0.86 (0.83–0.89)	0.89 (0.87–0.91)	0.87 (0.85–0.90)
*Time from TX to response, years*	4.6 (3.7–5.6)	5.0 (4.3–5.7)	4.8 (4.1–5.4)	4.6 (4.4–5.1)
*Diagnosis, in %*				
*DM*	26	27	14	22
*CGN*	15	31	28	15
*ADPKD*	15	14	28	26
*CIN*	11	4	6	8
*Nephrosclerosis*	4	6	2	7
*Other*	11	8	10	11
*Unknown*	19	10	12	10
*Education, in %*				
*basic education*	19	22	14	35
*high school*	19	24	40	31
*university*	44	55	38	18
*other*	15	0	4	10
*no data*	0	0	4	6
*Occupation, in %*				
*working*	44	51	58	19
*studying*	0	0	2	1
*unemployed*	15	2	2	4
*retired because CKD*	4	12	4	10
*retired (age)*	33	33	26	58
*other*	4	2	8	6
*Follow-up, years*	12.5 (11.3–13.7)	13.5 (12.7–14.3)	12.7 (11.7–13.7)	11.9 (11.1–12.8)
*Graft loss, in %*	33	25	36	43
*Patients’ death, in %*	12	22	22	37
*Death censored graft loss, in %*	7	16	22	11

The dialysis groups shared similarities in dialysis duration, age, primary kidney disease, comorbidity score (RRS), kidney function, and post-transplantation duration. Notably, HHD displayed a gender ratio difference (p = 0.021), with more men compared to other modalities (Table 1). Details regarding immunosuppression types are in S1 File.

Median meds numbered 11 (range 3–22), mean pill count was 14 (range 5–40), and mean MRCI totaled 15 (range 5–33). HRQoL didn’t correlate with medication quantity (R 0.66 p = 0.357), pill count (R 0.08 p = 0.257), or MRCI (R 0.83 p = 0.248). Pre-ICHD patients retired more often after transplantation (p = 0.009) and had higher mortality (p = 0.022) but similar graft survival (p = 0.370). Over 9931 CNI concentration results were examined (mean 49.6 times per participant, SD 21.6). ICHD patients had a higher CNI-COV (p = 0.011). In all, 59% had CNI-COV within 0–30%, with 80% of former HHD patients below this threshold vs. 43% of former ICHD patients (p<0.001). For PD groups, there were no differences. Detailed multimethod adherence assessments are in Table 2.

**Table 2:** Multimethod assessment of adherence split by previous dialysis modality. BAASIS- questionnaire results split by previous dialysis modality. VAS: visual analogue scale. COV: coefficient of variance; CNI: calcineurin inhibitor; MRCI: medication regimen complexity index. COV-CNI, pills, meds, and MRCI are values expressed in mean (95%CI) The rest of the results are in %. Subgroups with statistically significant differences are in bold.

ALL PARTICIPANTS	CAPD N = 27	APD N = 51	HHD N = 50	ICHD N = 72	p
COV for CNI	28. 1 (25.4–30.7)	29.8 (27.2–32.3)	**26.4 (23.4–28.5)**	**32.2 (29.4–34.9)**	**0.011**
COV for CNI < 30%	70%	55%	**80%**	**43%**	**0.001**
Pills #	15 (13–17)	13 (12–14)	16 (14–17)	15 (14–17)	0.095
Meds #	11 (10–13)	10 (9–11)	12 (11–12)	11 (10–12)	0.265
MRCI	16 (14–19)	15 (13–16)	16 (15–18)	16 (15–17)	0.394
**BAASIS**					
Prepare myself	96	98	98	96	0.941
Take myself	96	100	98	100	0.438
Missing doses					0.104
No	81.5	82.4	96	86.1	
Once	14.8	9.8	4.0	12.5	
Twice	0	5.9	0	1.4	
Thrice	0	2.0	0	0	
Four or more	3.7	0	0	0	
Timing					0.177
on time	59.3	54.9	70.0	75.0	
once later	18.5	17.6	16.0	9.7	
2–3 times later	11.1	19.6	10.0	9.7	
4–5 times later	0	3.9	2.0	4.2	
every 2-3days later	0	2.0	2.0	0	
almost every day	11.1	2.0	0	1.4	
Dose modification	0	0	0	0	1
Drug holidays	0	0	0	0	1
VAS, immunosuppression	89	94	92	84	0.138
VAS, other meds	88	94	91	83	0.100
**Q-SORT (N = 72)**					
	CAPD N = 10	APD N = 24	HHD N = 19	ICHD N = 19	p
Profile 1	40%	54%	47%	47%	
Profile 2	30%	29%	21%	32%	0.921
Profile 3	30%	17%	32%	21%	

### Non-adherence assessed with BAASIS

All but one patient prepared their pillboxes themselves and were able to take the medications without assistance. In the month preceding the study, 13.4% of patients missed at least one dose of immunosuppression, while 34% didn’t take medication on time. Details on non-adherence differences using the BAASIS questionnaire are in Table 2. Overall, no statistically significant differences in post-KT immunosuppression adherence were found regarding previous dialysis modalities. This trend persisted even when categorizing responses as "always on time vs. sometimes late" (p = 0.097) or "never missing a dose vs. sometimes missed" (p = 0.149). Patients’ perceived adherence on the visual analog scale exceeded the reported timing adherence, with a rate of over 84% for performance in the last 4 weeks.

Those who consistently took medications without missing doses and adhered to the schedule were older (mean age 58 vs. 55; p = 0.040) and had better HRQoL (mean 15D score 0.89 vs. 0.85; p = 0.023). Comorbidity scores (RRS score 4.2 for never missed vs. 4.0 for once or more missed; p = 0.07) and dialysis duration (26 months for never missed vs. 24 months; p = 0.545) didn’t differ significantly. Higher education level correlated with lower treatment adherence: 49% of university-level, 30% of high-school-level, and 17% of basic-level education patients showed nonadherence (p = 0.042).

### Patient attitudes toward transplantation

Out of the 101 participants who completed the Q-sort, 94 were properly filled out, and among these, 72 patients could be categorized into three distinct patterns of answers, each corresponding to a specific personality type. The remaining 22 did not align with any pattern. Factor analysis-derived Z-scores defined three profiles (profile 1, 2, and 3; S1 File), with 35 patients in profile 1, 20 in profile 2, and 17 in profile 3. Table 3 lists statements differentiating these profiles based on personality types. These profiles were equally distributed across subgroups categorized by previous dialysis mode (see Table 2).

**Table 3:** Q-sort statements are grouped according to personality type. The statements that statistically significantly distinguish all the profiles in different directions are shown in bold.

*Profile 1- efficient*, *organized*, *consistent*, *friendly*, *resilient* #4: I am afraid of dying due to a complication related to the transplant (disagreement) #5: I take all the medication as doctors say when the visit to the outpatient clinic is coming soon (disagreement) #6: My appearance changed because of my meds (neutral) #23: I always understand the doctor’s instructions (agreement) #29: I don’t experience side effects from the medications (agreement) #33: More information should be given to the patients before receiving a transplant (agreement) **#15: I can do everything I want, even though I have a transplant (agreement)** **#18: I am worried that my kidney will be rejected (neutral)** **#25: I would rather die than go back to dialysis ever again (disagree)** **#27: It is more difficult to get a job when you have a transplant (trend to agreement)** **#35: If I feel sick, I may change the dose of a medication or skip a dose (disagree)** **#37: I never forget my medication (agreement)**
*Profile 2- inventive*, *careless*, *outgoing* #7: Sometimes I may choose to take only the pills that are more important (neutral) #13 If I’m not sure whether I have taken my pills already, I take them again (neutral) #16: If you forget your immunosuppressive medication occasionally, nothing bad will happen (agreement) #24: Patients dialyzed at home are more adherent to the medication after transplantation (neutral) #26: On holidays it is easier to forget to take medications on time (agreement) #32: I find it difficult to tell my doctor about the side effects of the medications (neutral) #38: I would never go through the transplant process again (neutral) **#15: I can do everything I want, even though I have a transplant (neutral)** **#18: I am worried that my kidney will be rejected (trend to agreement)** **#25: I would rather die than go back to dialysis ever again (less disagreement)** **#27: It is more difficult to get a job when you have a transplant (neutral)** **#35: If I feel sick, I may change the dose of a medication or skip a dose (trend to neutral)** **#37: I never forget my medication (neutral)**
*Profile 3- challenging*, *cautious*, *organized*, *nervous/sensitive* #1: I do what doctors tell me; they know what the best for me is (neutral) #2: It is more important to be compliant than to enjoy life (disagreement) #8: I deserve more attention from my family because of my condition (neutral) #9: I cannot complain about the side effects of the drugs because I would be disgraceful (disagreement) #11: I would like to meet another transplant patient #17: I´m happy with my new kidney (neutral) #21: I would feel more secure if my transplant is controlled more often (agreement) #34: I don´t want my life to revolve around my disease (agreement) #36: I have problems swallowing larger pills (agreement) **#15: I can do everything I want, even though I have a transplant (disagreement)** **#18: I am worried that my kidney will be rejected (agreement)** **#25: I would rather die than go back to dialysis ever again (disagree the most)** **#27: It is more difficult to get a job when you have a transplant (agreement)** **#35: If I feel sick, I may change the dose of a medication or skip a dose (less disagreement)** **#37: I never forget my medication (disagreement)**
*Consensus statements* #3: My appearance is not very important to me (neutral) #10: I rather not tell others that I have a transplant (neutral) #12: I receive enough support from my friends and family (agreement) #14: If I do something somehow unhealthy, I tend to feel guilty (neutral) #19: I would not tell my doctor if I forgot to take my medication (disagreement) #20: For most of the medications, I don’t know why I take them (disagreement) #22: It is harder to remember taking the night dose of my medication (neutral) #28: Pillbox is a handy aid (agreement) #30: When I am out from home, I am not very punctual with my medication (disagreement) #31: I don’t mind the scars from transplant surgery (disagreement)

**Fig 3:**
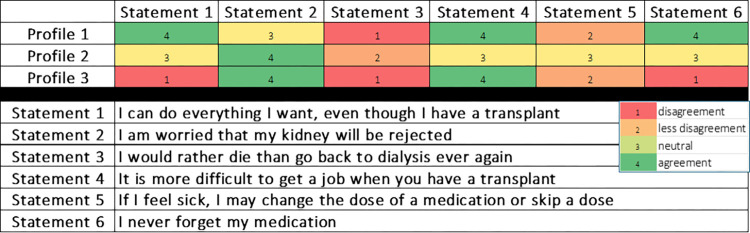
Distinction of the patient´s profiles based on six statements.

Among the statements, eight showed consensus among participants, while six statements played a key role in profile distinction (bold in Table 3). Analysis coupled with open comments from the Q-sort revealed that profile 1 predominantly comprised patients with a comprehensive understanding of their treatment, minimal complaints, and unrestricted daily life due to their transplant. Profile 2 consisted of more laid-back patients satisfied with their transplant but less inclined to fully adapt their lives to treatment requirements. Profile 3 encompassed patients worried about the future and struggling with medication side effects. Fig 3 A shows a graphical representation of six distinctive statements. Key differentiators between these three profiles were the number of meds, treatment complexity, and patient education (refer to Table 4).

**Table 4:** Differences between the three profiles were defined by Q-analysis. Results are expressed as means (95% CI). COV-CNI: coefficient of variance of calcineurin inhibitors. RRS: recipient risk score (i.e. comorbidity score). HRQoL: health-related quality of life. MRCI: medication regimen complexity index. *HHD, CAPD, APD, ICHD. Statistically significant differences are shown in bold.

	*Profile 1*	*Profile 2*	*Profile 3*	*p*
*Age at response, years*	58 (54–61)	58 (52–64)	61 (56–66)	0.525
*eGFR at response*	57 (50–65)	46 (36–56)	49 (35–62)	0.164
*Time spent on dialysis, months*	26 (21–32)	19 (13–26)	19 (12–27)	0.270
Type of dialysis, % *	26;11;37;26	20;15;35;30	35;18;24;23	0.921
*RRS*	4.3 (3.9–4.6)	4,1 (3.6–4.6)	4.3 (3.7–5.0)	0.831
*HRQoL*	0.87 (0.83–0.91)	0.82 (0.77–0.87)	0.85 (0.79–0.89)	0.177
*COV-CNI*	28 (25–32)	29 (25–32)	27 (23–31)	0.883
*Number of pills*	15 (13–16)	15 (12–17)	18 (15–22)	0.081
*Number of meds*	11 (10–12)	11 (9–12)	14 (12–15)	**0.012**
*MRCI*	16 (14–18)	14 /12-17)	20 (17–23)	**0.011**
*Education level*				**0.011**
*basic education*	**83**	17	**0**	
*high school*	54	**11**	**35**	
*university*	33	37	30	
*other*	29	25	0	

**Fig 4:**
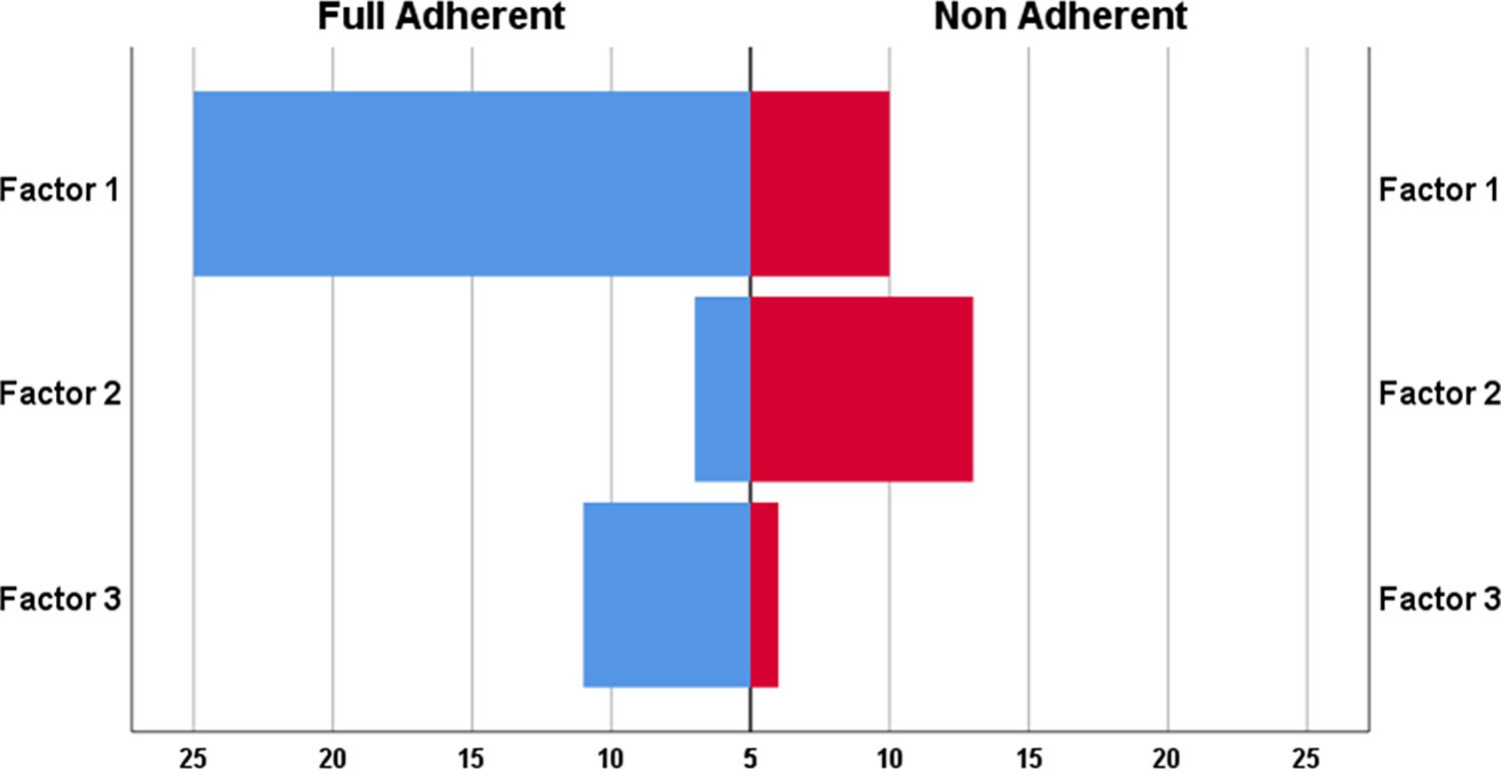
Fully adherent means never missing a dose and always taking the medication on time.

Notably, patients consistently adhering to their medication schedule without missing doses or timing (fully adherent) were predominantly in profile 1 (p = 0.029) (Fig 4)

### The associations between non-adherence and patients’ attitudes with graft survival

**Fig 5:**
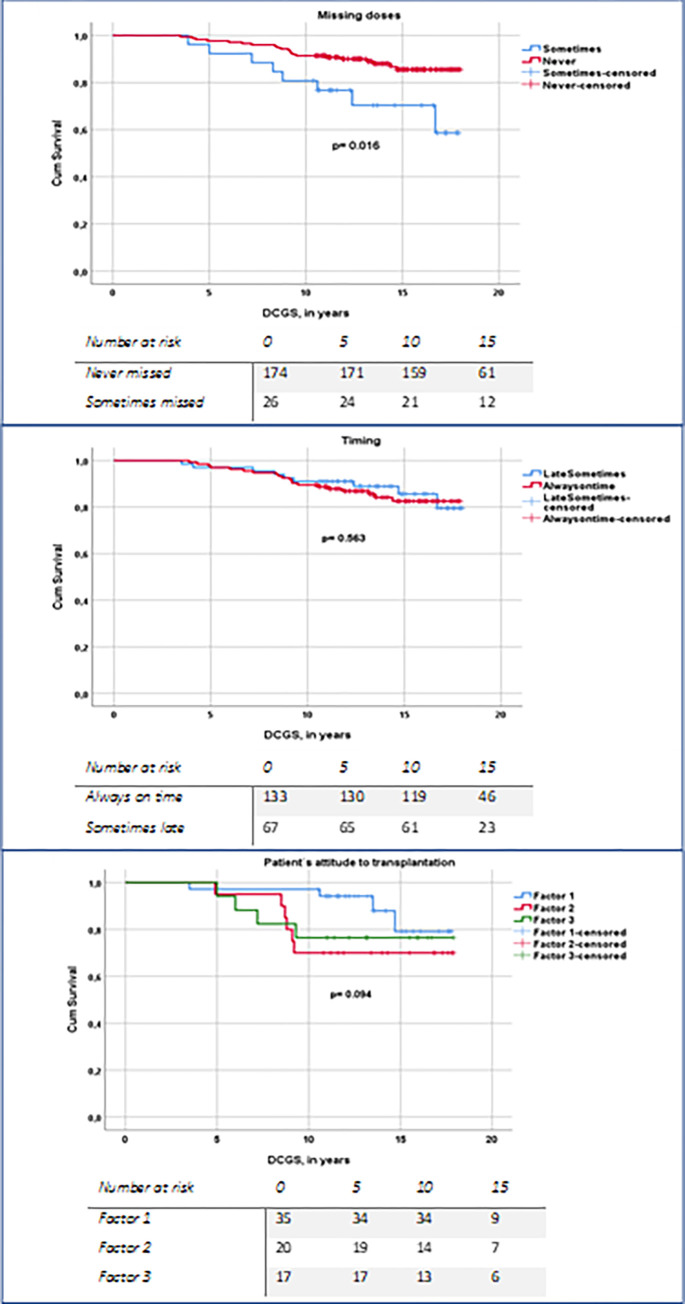
Death-censored graft survival (DCGS) curve in considering taking (upper panel), timing (middle panel), and patient attitudes to transplantation (lower panel) revealed by factor analysis showing the associations of the three profiles with DCGS.

The variability in timing and missing doses was most significant among participants. Missing a single immunosuppressant medication dose in the past four weeks correlated with lower death-censored graft survival (p = 0.016) (Fig 5). However, timing wasn’t associated with graft survival (p = 0.563). Deviations in timing or missing doses showed no difference between patients with or without acute rejection (p = 0.442 and p = 0.951).

Within the Q-sort analysis subgroup, patients exhibiting an attitude aligned with profile 1 trended towards better DCGS, although this didn’t reach statistical significance (p = 0.094, Fig 5 lower panel). Within this subgroup, Cox regression analysis, adjusted for age at response, acute rejection, and HRQoL, indicated that missing any medication dose in the prior four weeks correlated with a four-fold risk of graft loss. A higher COV-CNI was associated with increased graft loss risk (Table 5). Moreover, each additional pill raised the risk of graft loss by 7.6%.

**Table 5:** Cox regression analysis. Outcome: death censored graft survival. Statistically significant confounders are in bold. HR: hazard ratio. Timing: always versus never on time. Missing: never (ref) versus sometimes. Timing: always on time (ref) vs never on time. Age: at participation. AR: previous acute rejection (no is the ref). Factor: Q-sort factor analysis group.

	*Sig*	*HR*	*95% CI of HR*
*Number of pills*	**0.006**	**1.076**	**1.02–1.13**
*Age*	0.379	0.974	0.94–1.01
*HRQoL score*	0.631	2.487	0.061–101
*COV-CNI*	**0.049**	**1.029**	**1.00–1.06**
*Timing*	0.252	0.590	0.24–1.45
*Missing*	**0.004**	**4.128**	**1.55–10.97**
*AR*	0.675	0.763	0.51–3.13
*Profile 1*	Ref		
*Profile 2*	0.379	0.597	.24–1.66
*Profile 3*	0.687	0.827	.47–1.96

## Discussion

Our study found that prior dialysis modality was not associated with adherence to immunosuppressive treatment post-KT assessed by BAASIS. Variability in CNI concentration was higher in previous ICHD patients suggesting poorer adherence. The results of the Q-sort analysis revealed that inventive, careless, and outgoing personalities were less adherent, while efficient, organized, consistent, friendly, and resilient types were more adherent. Dialysis modality was not, however, associated with personality type. Of the studied adherence and attitude factors number of pills, COV-CNI and missing a dose were associated with an increased graft loss risk.

Previous studies have not looked specifically at the association between dialysis modality and medication adherence. However, research from Canada found that HHD patients exhibited higher health literacy than non-dialysis and ICHD patients and low health literacy was associated with negative outcomes in CKD and lower kidney graft function [3]. Another Canadian study highlighted poorer health literacy being linked to lower immunosuppression adherence [22]. In the present study, both homedialysis modalities were included: PD and HHD patients. These results are different to compare, particularly because health literacy was not investigated in this study. However, the literacy level showed an association with adherence: PD patients had higher education, but they missed doses more frequently and struggled more with timely medication intake. Another difference in this study compared to others is the timing of questionnaire completion, possibly affecting health literacy changes over time. Namely, in our study the tool was applied once 4.7 years post-transplantation. Along these lines, the methodology applied in this research mainly evaluated the persistence of the immunosuppressive regimen. Recent research suggested that high health literacy didn’t necessarily translate to improved kidney graft survival [23], aligning with our findings. The absence of a gold standard for health literacy assessment in CKD patients challenges result comparisons. A multi-center randomized controlled trial investigating the impact of (video education plus behavior contract versus standard of care, only 58% adhere to the intervention and it did not improve any outcomes [24]. The authors reinforce the idea non-adherence is a difficult multifactorial problem requiring much more than simple solutions. Our hypothesis was that patients who received therapeutic education before transplantation during homedialysis training would be more adherent to immunosuppression after. We were unable to detect differences in the level of adherence in our patients despite the therapeutic education received. Similar results were observed in a cohort of French patients in which therapeutic education prior to transplantation did no improve adherence, although it lowered adverse effects reporting and improved HRQoL [25].

Personality traits are pivotal in assessing adherence in chronic disease individuals [10]. The results of the Q-sort analysis revealed that inventive, careless, and outgoing personalities were less adherent, while efficient, organized, consistent, friendly, and resilient types were more adherent. Most low-educated patients belonged to the latter profile, which showed a trend for better graft survival. Patients with a challenging personality expressed more medication concerns and were less satisfied with KT. This observation is consistent with the results from a scoping review on patient-related factors of medication adherence, where the authors highlighted the desire of the patients to discuss their concerns about medications, to expect more information on medicines, and to better communicate with healthcare professionals [26]. Adherence to immunosuppression is a complex concept determined by a myriad of factors that affect the outcomes [27]. Exploring the capabilities, opportunities, and motivation to adhere to medical treatment explains why the patient does not engage with the desired behavior and may be an intervention to improve adherence [28]. In this study six different statements differentiated patient profiles: “I can do everything I want, even though I have a transplant”, “I am worried that my kidney will be rejected”, “I would rather die than go back to dialysis ever again”, “It is more difficult to get a job when you have a transplant”, “If I feel sick, I may change the dose of a medication or skip a dose” and “I never forget my medication.” Further evaluation of these simple points of view could help healthcare professionals dig into the challenges of medication non-adherence.

Personality types and its association with non-adherence was investigated in a cohort of Dutch patients [20] While the research methodology we applied resembled that study, the Dutch authors found an association between patient attitudes and tacrolimus concentration variability but not with self-reported non-adherence or graft survival. In contrast, we did not find any link between personality type and COV-CNI, but we observed a connection between self-reported non-adherence and a trend toward poorer death-censored graft survival. The divergence in findings between these studies could be partly explained by differences in the number of drug concentration measurements (5 vs. 54), duration of follow-up (2 vs. 14 years), time elapsed from KT to response, lower incidence of graft failures, and patient mortality in the Dutch study. Additionally, while our BAASIS survey was answered anonymously, the Dutch study relied on interviews, potentially reflecting variations in patient expectations across different populations. A recent meta-analysis showed that BAASIS-assessed nonadherence was significantly associated with higher variability in tacrolimus concentrations (P = 0.02), higher barriers (P < 0.001), lower self-efficacy (P < 0.001), lower intention (P < 0.001), and higher worries (P = 0.02), among other variables. Our research agrees with these observations [29]. It is worth mentioning that our study design did not focus on initiation and the implementation of the prescribed regimen could not be corroborated with the pharmacy repository. According with the taxonomy for adherence, our study focused on persistence on the prescribed regimen evaluated with self-assessment. We intended to apply the tools that are easily available in the clinical setting.

In this study, neither HRQoL nor comorbidities demonstrated a contribution to non-adherence. Instead, factors such as pill burden, high COV-CNI, and missing immunosuppressive doses in the previous four weeks notably diminished graft survival. Although high tacrolimus intrapatient variability is linked to acute rejection and non-adherence, substantial evidence supporting this remains limited [30]. Interestingly, while COV-CNI significantly affected death-censored graft survival in this research, the mean COV-CNI was comparable between adherent and non-adherent patients. This discovery might be explained by a positive adherence phenomenon, indicating an improvement in patient adherence closer to their next physician appointment. The challenges related to pill burden and medication regimen complexity highlighted in our study align with existing literature [31], emphasizing the complexities of managing polypharmacy in KT. This problem particularly increases with age. Our patients were prescribed between 14 to 17 pills a day, and this is in accordance with a qualitative study performed in older patients [32]. To them the main issues emerged from the assessment were the variability in health literacy toward medicines, the importance of support networks, the need to adjust health expectations, motivators for self-care, personalized to medication taking and management. In our study we counted pill number from the prescription, but this did not ensure their intake or even if it was dispensed from the pharmacy. Other tool for measuring pill intake is the use of microprocessors embedded in the medication containers, although this has been mostly applied in research and difficult to expand to the clinical setting due to the higher costs [15].

Limitations include the lack of a specific kidney health literacy tool assessment, and the study’s cross-sectional nature restricted the assessment of non-adherence dynamics. Another limitation is that there was no assessment of pretransplant adherence. However, self-efficacy is a requirement for being on home dialysis and lack of adherence to dialysis and medical treatment is considered a contraindication for organ transplantation. The sample was highly variable in post-transplant time, which was on average 4.7 years. Medication adherence is a dynamic process, and this study thus mainly evaluated the persistence of the immunosuppressive regimen. The response rate was 62%, and non-respondents might differ in treatment attitudes. Exploration of patient behavior was limited due to Q-sort completion difficulties, affecting analysis power. Despite limitations, strengths lie in the long follow-up and comprehensive overview of non-adherence factors.

In conclusion, home dialysis didn’t correlate with improved immunosuppression adherence post-KT assessed by BAASIS, although previous ICHD patients had a higher COV-CNI suggesting challenges in medication scheduling. Higher education didn’t ensure better adherence to immunosuppressive medications. Skipping immunosuppressant doses, high pill count, COV-CNI over 30%, and six selected Q-sort statements contributed to identifying patients at risk of graft loss. All these tools could be easily adopted into clinical practice. This investigation raises the need for future research to prove if continuous monitoring of these variables in a larger cohort could improve kidney transplant outcomes.

## Supporting information

S1 Checklist Strobe check list. (PDF)

S1 Questionnaires (PDF)

S1 File Additional results. (PDF)
