# Supplementary material for: Associations between dialysis modality and adherence to immunosuppression after kidney transplantation—A single-center study
Source: PLoS One. 2025 Jan 24;20(1):e0317435. doi: 10.1371/journal.pone.0317435 (PMC11760586; doi:10.1371/journal.pone.0317435)
Supplement: S1 Checklist — (PDF) [file pone.0317435.s001.pdf]

STROBE Statement—checklist of items that should be included in reports of observational studies

	Item No.	Recommendation	Page No.	Relevant text from manuscript
**Title and abstract**	1	(*a*) Indicate the study's design with a commonly used term in the title or the abstract	2	cross sectional
		(*b*) Provide in the abstract an informative and balanced summary of what was done and what was found	2	
**Introduction**				
Background/rationale	2	Explain the scientific background and rationale for the investigation being reported	3	self efficacy and adherence
Objectives	3	State specific objectives, including any prespecified hypotheses	4	adherence is better in previous homedialysis patients
**Methods**				
Study design	4	Present key elements of study design early in the paper	4	cross sectional study in incidental kidney transplant patients
Setting	5	Describe the setting, locations, and relevant dates, including periods of recruitment, exposure, follow-up, and data collection	4	under the subtitle: Design, study setting, and participants
Participants	6	*Cross-sectional study*—Give the eligibility criteria, and the sources and methods of selection of participants	4	incidental kidney transplant patients from the outpatient clinic, adults
Variables	7	Clearly define all outcomes, exposures, predictors, potential confounders, and effect modifiers. Give diagnostic criteria, if applicable	5	Measurements
Data sources/ measurement	8*	For each variable of interest, give sources of data and details of methods of assessment (measurement). Describe comparability of assessment methods if there is more than one group	5-6	Measurement of adherence
Bias	9	Describe any efforts to address potential sources of bias	6<br>Fig 2	If patient found difficult to fill the survey, assistance was provided.<br>Directed acyclic graph
Study size	10	Explain how the study size was arrived at	Fig 1	Flow chart

Continued on next page

Quantitative variables	11	Explain how quantitative variables were handled in the analyses. If applicable, describe which groupings were chosen and why	6	Previous dialysis mode was used according to the hypothesis
Statistical methods	12	(*a*) Describe all statistical methods, including those used to control for confounding	6-7	
		(*b*) Describe any methods used to examine subgroups and interactions	6-7	
		(*c*) Explain how missing data were addressed	Fig 1	Flow chart
		(*d*) C*ross-sectional study*—If applicable, describe analytical methods taking account of sampling strategy	NA	
		(*e*) Describe any sensitivity analyses	7	
**Results**				
Participants	13*	(a) Report numbers of individuals at each stage of study—eg numbers potentially eligible, examined for eligibility, confirmed eligible, included in the study, completing follow-up, and analysed	Fig 1	All these items are explained in the figure
		(b) Give reasons for non-participation at each stage		
		(c) Consider use of a flow diagram		
Descriptive data	14*	(a) Give characteristics of study participants (eg demographic, clinical, social) and information on exposures and potential confounders	7 – Table 1	
		(b) Indicate number of participants with missing data for each variable of interest	Fig 1	
		(c) *Cohort study*—Summarise follow-up time (eg, average and total amount)		
Outcome data	15*	*Cross-sectional study*—Report numbers of outcome events or summary measures	Table 1 & 2	
Main results	16	(*a*) Give unadjusted estimates and, if applicable, confounder-adjusted estimates and their precision (eg, 95% confidence interval). Make clear which confounders were adjusted for and why they were included	8-10<br>Tables 3 & 4	
		(*b*) Report category boundaries when continuous variables were categorized	Table 2	
		(*c*) If relevant, consider translating estimates of relative risk into absolute risk for a meaningful time period		Not applicable

Continued on next page

Other analyses	17	Report other analyses done—eg analyses of subgroups and interactions, and sensitivity analyses	Table 5	Cox regression
**Discussion**				
Key results	18	Summarise key results with reference to study objectives	10	First paragraph
Limitations	19	Discuss limitations of the study, taking into account sources of potential bias or imprecision. Discuss both direction and magnitude of any potential bias	14	
Interpretation	20	Give a cautious overall interpretation of results considering objectives, limitations, multiplicity of analyses, results from similar studies, and other relevant evidence	11-13	
Generalisability	21	Discuss the generalisability (external validity) of the study results	14	Last paragraph
**Other information**				
Funding	22	Give the source of funding and the role of the funders for the present study and, if applicable, for the original study on which the present article is based		

*Give information separately for cases and controls in case-control studies and, if applicable, for exposed and unexposed groups in cohort and cross-sectional studies.

Note: An Explanation and Elaboration article discusses each checklist item and gives methodological background and published examples of transparent reporting. The STROBE checklist is best used in conjunction with this article (freely available on the Web sites of PLoS Medicine at http://www.plosmedicine.org/, Annals of Internal Medicine at http://www.annals.org/, and Epidemiology at http://www.epidem.com/). Information on the STROBE Initiative is available at www.strobe-statement.org.
