# Supplementary material for: Associations between dialysis modality and adherence to immunosuppression after kidney transplantation—A single-center study
Source: PLoS One. 2025 Jan 24;20(1):e0317435. doi: 10.1371/journal.pone.0317435 (PMC11760586; doi:10.1371/journal.pone.0317435)
Supplement: S1 Questionnaires — (PDF) [file pone.0317435.s002.pdf]

Annex A: Education and employment status

Patient identification number: _____

A1- Education before transplantation

a. basic education

b. high school graduated

c. advanced degree

d. other (Specify)

A2- Employment status

2.1. Where were you employed before transplantation? Yes / No

If the previous answer was No choose one of following options:

a- retired

b-studying

c-unemployed

d-early retired for a cause associated with my kidney disease

e-early retired for another cause

f-other (specify)

2.2. Are you currently employed? Yes/ No

If the previous answer was No, choose one of following options:

a- retired

b-studying

c-unemployed

d-early retired for a cause associated with my kidney disease

e-early retired for another cause

f-other (specify)

Annex B: Instructions for the Q-method survey

Patient identification number: ____

INSTRUCTIONS FOR THE SURVEY

These instructions will guide you through the survey step by step. Please read each step to the end before you start carrying it out.

1. Take the deck of cards [see Annex D-1] and the score sheet and go sit at a table. Lay down the score sheet [see Annex D-2] in front of you. All 38 cards in the deck contain a statement concerning kidney transplantation. We will ask you to rank-order these statements from your point of view. Our question to you is: “To what extent do you agree with the following statements”. The numbers on the cards (from 1 to 38) have been assigned to the cards randomly and are only relevant for the administration of your response.

2. This study is about patients’ behavior. We are interested in **your attitude towards kidney transplant treatment**.

3. Read the 38 statements carefully and split them up into three piles: a pile for statements you tend to disagree with, a pile for cards you tend to agree with, and a pile for cards you neither agree nor disagree with or that are not relevant or applicable to you. Please use the three boxes “AGREE”, “NEUTRAL OR NOT RELEVANT” and “DISAGREE” at the bottom left of the score sheet. Just to be clear, we are interested in your point of view. Therefore, there are no right or wrong answers. When you have finished laying down the cards in the three boxes on the score sheet, count the number of cards in each pile and write down this number in the corresponding box. Please check whether the numbers you entered in the three boxes add up to 38.

4. Take the cards from the “AGREE” pile and read them again. Select the two statements you most agree with for kidney transplant treatment and place them in the two last boxes on the right of the score sheet, below the “9” (it does not matter which one goes on top or below). Next, from the remaining cards in the deck, select the three statements you most agree with and place them in the three boxes below the “8”. Follow this procedure for all cards from the “AGREE” pile.

5. Now take the cards from the “DISAGREE” pile and read them again. Just like before, select the two statements you most disagree with and place them in the two last boxes on the left of the score sheet, below the “1”. Follow this procedure for all cards from the “DISAGREE” pile.

6. Finally, take the remaining cards and read them again. Arrange the cards in the remaining open boxes of the score sheet.

7. When you have placed all cards on the score sheet, please go over your distribution once more and shift cards if you want to.

8. Please explain why you agree most with the two statements you have placed below the “8”.

Card number	
Card number	

9. Please explain why you disagree most with the two statements you have placed below the “1”.

Card number	
Card number	

Annex B-1: 38 statements

17-I am happy with my new kid-ney	18-I am worried that my kidney will be rejected	25-I would rather die than go back to dialysis ever again
3-My appearance is not very important to me	15-I can do everything I want, even though I have a transplant	10-I rather not tell others that I have a transplant
1-I do what doctors tell me, they know what is the best for me	31-I don't mind the scars from the transplant surgery	29-I experience no-side effects from the medications
2-It is more important to enjoy a life that to be compliant	24-Patients dialyzed at home are more adherent to the medica-tion after transplantation	30-When I am out from home, I am not very punctual with my medication
28-A pillbox is a handy aid	21-I would feel more secure if my kidney function is monitored more often	34-I do not want my life to re-volve around my disease
14-If I do something that is not so healthy, I tend to feel guilty	16-If you occasionally forget your immunosuppressive medi-cation nothing really bad will happen	37-I never forget my medication
19-I would not tell my doctor if I forgot to take my medication	6- My appearance changed be-cause of the immunosuppressive medication	20-For most of the medication, I don't know why I take it
12-I receive enough support from my friends and family	13-If I am not sure whether I have already taken my pills, I just take them again	5-I take all the medication as doctors say when the visit to the outpatient clinic is coming soon
35-If I feel sick, I may change the dose of a medication or skip a dose	38-I would never go through the transplant process again	7-Sometimes I choose to take only the more important pills
22-It is harder to remember to take the night dose of my medi-cation	26-On holidays it is easier to forget to take the medications on time	8-I think I deserve more atten-tion from my family because of my disease
23-I always understand the doc-tor's instructions	36-I have problems swallowing larger pills	11-I would like to meet other transplant patients
27-It is more difficult to get a job when you have a transplant	32-I find it difficult to tell my doctor about the side effects of the medications	9-I think I cannot complain about the side effects of the drugs because it would be dis-graceful
33-More information should be given to the patients before receiving a transplant	4- I am afraid of dying due to a complication related to the transplant	

Annex B-2: Score sheet for Q sorting

Patient identification number: ____

**DISAGREE**<br><br>**COUNT: ___**	**NEUTRAL OR**<br>**NOT RELEVANT**<br><br>**COUNT: ___**	**AGREE**<br><br>**COUNT: ___**

disagree most **agree most**

**1**	**2**	**3**	**4**	**5**	**6**	**7**	**8**	**9**

Annex E: Medication Regimen Complexity Index (to be filled in by the research nurse)

Patient identification number: ____

Dosing frequency	Medications												Total	Weighting	Weighting x number of medications
Once daily														1	
Once daily prn														0,5	
Twice daily														2	
Twice daily prn														1	
Three times daily														3	
Three times daily prn														1,5	
Four times daily														4	
Four times daily prn														2	
q 12 h														2,5	
q 12 h prn														1,5	
q 8 h														3,5	
q 8 h prn														2	
q 6 h														4,5	
q 6 h prn														2,5	
On alternate days or less frequently														2	
