# Supplementary material for: Associations between dialysis modality and adherence to immunosuppression after kidney transplantation—A single-center study
Source: PLoS One. 2025 Jan 24;20(1):e0317435. doi: 10.1371/journal.pone.0317435 (PMC11760586; doi:10.1371/journal.pone.0317435)
Supplement: S1 File — (PDF) [file pone.0317435.s003.pdf]

Supplemental material S2– Additional Results

Comparison of medical treatment regarding previous dialysis modality

	CAPD (N=27)	APD (N= 51)	HHD (N= 50)	ICHD (N=72)	P
Immunosuppression					
Cyclosporin A	66	59	66	68	0.752
Tacrolimus TD	15	8	16	13	0.636
Tacrolimus LR	19	31	18	17	0.213
MMF	100	84	88	83	0.153
Azathioprine	0	6	0	1	0.128
Corticosteroids	7	2	6	16	0.137

Immunosuppressive use, expressed in %. TD: twice daily. LR: long release. MMF: mophetil mycophenolate. m-Tor inhibitor users were omitted due to the low number of cases.

Q-sort Z-scores distribution

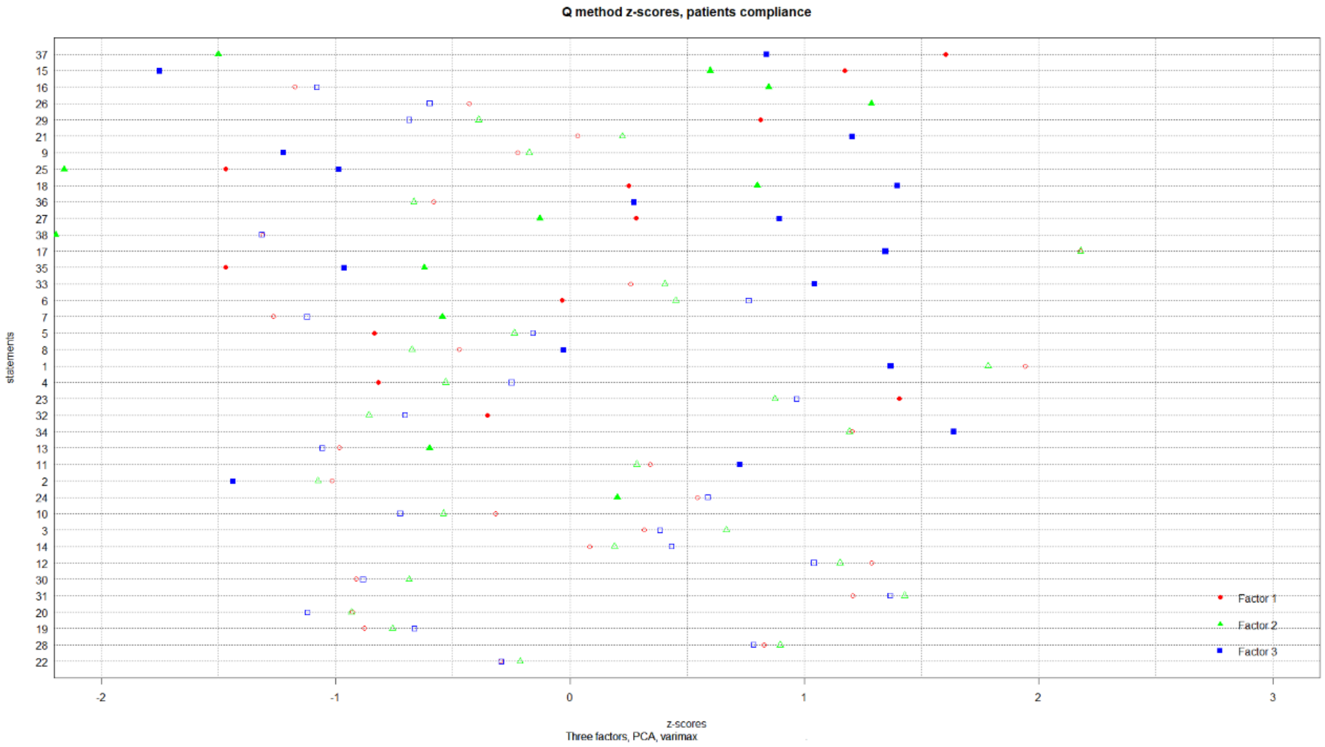

In the Y-axis the statement number (content is displayed in Table 3 of the manuscript. In the X-axis the distribution of Z-scores from -2 to +2.
